# The Design and Implementation of a Phased Antenna Array System for LEO Satellite Communications

**DOI:** 10.3390/s24061915

**Published:** 2024-03-16

**Authors:** Cezar-Ion Adomnitei, Cezar-Eduard Lesanu, Adrian Done, Ang Yu, Mihai Dimian, Alexandru Lavric

**Affiliations:** 1 Department of Computers, Electronics and Automation, Stefan cel Mare University of Suceava, 720229 Suceava, Romania; 2 Integrated Center for Research, Development and Innovation in Advanced Materials, Nanotechnologies and Distributed Systems for Fabrication and Control, Stefan cel Mare University of Suceava, 720229 Suceava, Romania; 3 Electrical Engineering and Computer Science, Carolina University, Winston-Salem, NC 27101, USA

**Keywords:** antenna radiation pattern, beam steering, electronic phase control, LEO satellite communications, phased antenna array

## Abstract

LEO satellite constellations can provide a viable alternative to expand connectivity to remote, isolated geographical areas and complement existing IoT terrestrial communication infrastructures. This paper aims to improve LEO satellite communications by implementing a new phased antenna array system that can significantly improve the radio communication link’s performance. By adjusting the progressive phase shift to each element of the antenna array system, the direction of the main radiation lobe of the phased antenna array system can be controlled with accuracy. As far as we know, it is the first time that a four-element, three-quarter wavelength phased antenna array system has been successfully realized with the intention of being optimized for implementation in LEO IoT satellite reception systems. The proposed system’s high level of performance is confirmed by the measurements, which indicate effective control of the main radiation lobe orientation. The numerical analysis shows a maximum gain close to 12 dBi for about 42° elevation, a Half Power Beamwidth (HPBW) of 32° in the vertical plane, and 80° in the azimuth plane. The experimental measurement results at various main lobe orientation angles revealed an HPBW ranging from 76° to 87° in the azimuth plane and a maximum Front-to-Back ratio (F/B) of 14.5 dB.

## 1. Introduction

During the past years, the satellite communications market is expected to reach USD 160.65 billion by 2030 [1] and will continue to grow in the next years, sustained by the development of new technologies. The number of applications of satellite communications extended NB-IoT (Narrow Band Internet of Things) connectivity from space to remote, isolated geographical areas. The number of LEO (Low Earth Orbit) satellites gained immense popularity due to some distinct advantages in satellite communications, which can compensate for the shortcomings associated with communications using GEO (Geostationary Earth Orbit) and MEO (Medium Earth Orbit) satellites. The main advantages include reduced latency due to short distances between satellites and Earth, improved global coverage given orbital trajectories, and the flexibility and scalability of LEO systems that can quickly adapt to user requirements by adding or reconfiguring existing satellites. These features, along with the extensive use of multiple frequency bands, help improve performance in congested environments such as those found in various IoT architectures. Additionally, ensuring a greater immunity to interference and oversaturation of radio frequencies by using a large number of satellites with the availability of several frequency bands can lead to better performance in congested radio communications scenarios specific to different IoT architectures.

Moreover, the latency associated with GEO satellite communications creates problems in latency-sensitive applications such as real-time communications (video conferencing), financial transactions, applications that include Augmented Reality (AR) and Virtual Reality (VR), Internet of Things (IoT), medical telemedicine, and autonomous transportation. Therefore, GEO satellites are mainly used for one-way communications, such as satellite television, monitoring of Earth’s natural resources, and other reduced latency-sensitive applications [2].

The above performance aspects, as well as the increasing requirements for enhanced transfer rates via increased data throughput efficiency and fast global coverage, have been the determining factors for industry-leading companies to invest in the development of large LEO satellite constellations. The main objectives behind these initiatives include providing fast and secure communication services and high-speed Internet, including in rural, isolated areas or with poor data connectivity, remote observations, and navigation. Companies such as SpaceX with the Starlink [3] constellation, OneWeb (Oneweb Network Access Associates Limited—London [4]), Amazon with the Kuiper project [5], and the Hongyan constellation developed by China Aerospace Science and Technology Corporation (CASC) [6] have invested significantly in the development and launch of LEO satellite constellations. The frequency bands used by LEO constellations start from VHF (30–300) MHz and UHF bands (300 MHz–3GHz) and continue to Ku-band (12–18) GHz, Ka-band (26.5–40) GHz and V-band (40–75) GHz.

However, there are certain disadvantages associated with using these LEO satellite constellations. The large number of satellites required for a large geographical coverage (hundreds or even thousands of satellites) can lead to very high costs and risks of collision with other satellites or space debris. Additionally, compared to GEO satellite communications, LEO satellite communications can be affected by the interference caused by the general state of the weather due to the lower altitude, and on the other hand, the latency can vary depending on the location of the user or the ground station. Last but not least, there is the need for an extensive network of ground stations, including antennas or antenna systems that can track satellites throughout their entire orbit.

Despite these drawbacks, LEO constellations remain an attractive option in the field of satellite communications, providing a viable alternative to complement terrestrial communications infrastructure to expand the connectivity area. This paper explores the improvement in LEO satellite communications by defining and implementing a phased antenna array system based on an original concept that can significantly increase the performance level of the radio communication link.

The UHF band from 435 MHz to 438 MHz offers a distinct set of features and advantages that make it suitable for certain applications and services [7,8]. Among those, communications with CubeSat satellites [9] (specific size and construction standard for small satellites), picosatellites [10], and other small satellites allow receiving images, environmental monitoring, reception of data collected from sensors located on the satellite (data from space or from the environments through which the satellite passes during its orbit) or data collected using sensors located on Earth (e.g., IoT applications). The 435–438 MHz frequency band is used for data collection in terrestrial, atmospheric, or aquatic environments where the IoT sensors located on the ground transmit the data to the satellites. In this paper, we will focus on a phased antenna array system designed for the 435–438 MHz frequency band used by LEO satellite communications.

This article brings a new concept in the field of phase antenna arrays via the type and number of elements used and their implementation in a compact system, using a consistent ground plane that integrates all the hardware components necessary for correct operation. The potential applications and significance of a four-element square-phased antenna array system that can enhance the performance level of LEO satellite communications are explored.

Following the introduction, Section 2 provides a concise overview of common satellite communication antennas, the current landscape of phased antenna arrays, and their underlying technologies for LEO satellite communications. Additionally, it provides a brief overview of the proposed phased antenna array system and its conceptual originality. Section 3 has the purpose of clarifying certain theoretical considerations on the rectangular planar antenna array radiation pattern and the derivation of the phase shifts required for the elements’ feeding signals in the case of the proposed four-element square-phased antenna array. Section 4 shows the general operation principle of controlled reception of a certain LEO satellite using the proposed phased antenna array system and illustrates the potential of the proposed system to be implemented in such a specific application. Section 5 furnishes the design and implementation details of the proposed system, while Section 6 outlines the experimental methods and procedures employed in acquiring the array’s radiation pattern and offers a comparative analysis alongside the limitations of the proposed system. Section 7 emphasizes conclusions and discusses future work using the proposed system in LEO satellite communications.

## 2. Antenna Design Challenges for Leo Satellite Communications

The types of antennas used in GEO satellite reception are usually parabolic antennas of various sizes, which meet specific gain and bandwidth requirements. The wide range of MEO satellites orbiting altitudes led to the need to use a variety of antenna types, depending on the applications pursued; among these types worth mentioning are parabolic, patch (flat), helix (spiral), Yagi (directives), and other types of antennas. To receive signals from LEO satellites in the 435–438 MHz frequency band, several types of antennas are commonly used, including Yagi, Helix, patch, and SDR (Software-Defined Radio) reception antennas.

Although this frequency band was initially allocated (1930s) for radio amateurs, with the beginning of modern technological development (1960s–1970s), interest in the use of outer space for communication and research purposes increased. Both radio amateurs and researchers began to use this frequency band for communications with research satellites and conducting experiments in the space field, which include the use of data from various types of sensors mounted on satellites, various experiments like communications with space crews, microgravity, navigation, and others). Starting with the year 2000, the standardization and development of small satellites (CubeSat, PocketQube, and others) led to the current use of this frequency band for communications with these research LEO satellites.

The Yagi antennas, due to their remarkable gain and directivity, are commonly employed to achieve expansive azimuthal coverage and track Low Earth Orbit (LEO) satellites to maintain reception integrity. Mechanical systems, commonly referred to as rotators, are used to facilitate this functionality. These rotator systems have many disadvantages, like damage due to rapid wear of the mechanical elements, damage caused by environmental conditions, and the need for frequent and regular maintenance, which leads to low performance in poor weather conditions.

With the intention of mitigating or overcoming these disadvantages, alternative versions based on different concepts were explored and experimented with. Among these breakthroughs is the development of phased antenna arrays [11,12,13,14,15,16], which emerged as a game changer in the field of satellite communications. Due to their characteristics and the advantages offered, phased antenna arrays can represent successful candidates for implementation in future specific ground stations for LEO satellite communications. A phased antenna array is a compact system of antennas whose directivity can be controlled electronically without requiring mechanical elements to change their position. That is why it can be called a static antenna with electronically controllable directivity. In a phased antenna array, the radiating elements can be arranged in certain topologies, depending on the specifics of the application, and using various methods of controlling the phase and/or amplitude of the feeding signals of each element, the maximum radiation can be oriented in the desired direction.

One of the proposed projects of the Libre Space Foundation aimed to replace the rotator-based antennas, already in operation within distributed earth stations mentioned in [17,18], with four-element helix phased antenna arrays to improve LEO satellites reception and, at the same time, provide the advantage of electronic beam steering control, without the need for moving mechanical components. On the other hand, the creation of a four-element helix phased antenna array involves many variables that are very difficult to manage, starting from the design until bringing it into operation. Another approach is presented in [19], which addresses the use of delay lines as a method of achieving phase shifts between the feeding signals of the antenna array elements. This solution for phase shifters brings the disadvantage of a reduced angular resolution and increased complexity of the delay line networks, even if the computing power requirement is not very high. Advantages offered by new technologies based on coherent SDR receivers [20], such as the KrakenSDR project [21,22], offers a high angular resolution and the capability of simultaneous multi-satellite tracking but requires very high processing power, high system complexity, and consequently long delays. Although this technology allows for very interesting applications, such as radio direction finding, it can also be used for tracking and receiving LEO satellites.

The medium elevation range (25–70°) is often preferred in LEO satellite communications for several reasons. High altitude passes (70–90°) are rare and brief, with stronger Doppler effects due to maximum orbital speeds. Low elevation passes (0–25°) have longer durations but suffer from atmospheric absorption, interference, and reflections, particularly at greater distances. At medium elevations, approximately 35% of total transit time, the signal remains more intact with lower losses and less pronounced Doppler effects. Consequently, we proposed the implementation of the 3λ/4 configuration for the antenna array elements, as it inherently exhibits favorable radiation characteristics within this elevation range, enabling the concentration of maximum radiated energy towards this angular interval.

The three-quarter wavelength (3λ/4) antenna element offers several advantages that will be highlighted in the following paragraphs. Different studies [23,24,25,26] revealed that compared to other antenna configurations, such as half-wavelength (λ/2) and the widely used quarter-wavelength (λ/4), in the case of the 3λ/4 antenna element, in the radiation pattern’s vertical plane, the maximum radiation lobe (main lobe) rises towards medium elevation angles, while preserving its omnidirectional characteristic in the horizontal plane. Additionally, even at low elevation angles, the 3λ/4 antenna gain is superior to, or at least equal to, that offered by the widely used conventional λ/4 vertical monopole elements, but also to that provided by the λ/2 configuration. Furthermore, 3λ/4 vertical monopole elements used together with at least two radial elements arranged symmetrically with respect to their feeding points or used in the variant where they are fixed to a consistent metallic ground plane maintain the impedance at a value very close to 50 Ohms, regardless of the resonant frequency they are tuned to. The physical implementation is relatively simple, and the procedures for tuning those antenna elements and fixing them on the ground plane are not overly complex. Another advantage is related to the radiation pattern in the vertical plane remaining favorable for medium elevation angles. The design, implementation, and testing of such a complex system requires advanced knowledge in the field of antennas and communications.

A phased antenna array, based on an original conceptual approach, through incorporating three-quarter wavelength elements and introducing an innovative constructive concept regarding the structuring of other necessary elements for array operation, is proposed in this article. In general, achieving beam steering using a controlled phase-shifting technique in phased antenna array systems is not new, as it has been recognized and implemented in various contexts, including industrial applications. In this paper, the focus was not on the development of advanced beamforming techniques involving the control of both amplitudes and phases using complex mathematical beamforming algorithms. Instead, the primary objective was to demonstrate the system’s functionality as a holistic concept in terms of the ability to direct the main radiation lobe in the desired direction, an essential feature for tracking LEO (Low Earth Orbit) satellites. The known performances of the AD8340 and DAC8554 circuits, incorporated in the phase shifters used, have been taken into account and are available in the technical specifications of these circuits [27,28]. As far as we know, it is the first time that a four-element, three-quarter wavelength phased antenna array system has been successfully realized with the intention of being optimized for implementation in LEO satellite reception systems. Regarding the hardware perspective of the proposed antenna array system, the conceptual approach was delineated by the distinctive features of the phased antenna system, including the integration of active hardware components, such as phase shifters and their controller, into a unified module. This allows serial communication with various computing systems (such as PCs, Raspberry Pi, or other SBCs) via a USB serial interface. These components are integrated onto a single solid PCB, mechanically fixed, electromagnetically shielded, and grounded. The signal adder is directly connected to the phase shifters via identical short-length professional connectors, which do not introduce additional attenuation or phase shifts, thus obviating the need for delay lines that could induce significant phase shifts. Furthermore, all hardware is positioned below the ground plane to minimize interference and external electromagnetic influences. The use of appropriate simulations and tests was essential to verify the actual performance of the proposed array.

## 3. Theoretical Considerations on Phased Antenna Arrays for Leo Satellite Communications

### 3.1. Phased Antenna Arrays for LEO Satellite Communications

**Figure 1:**
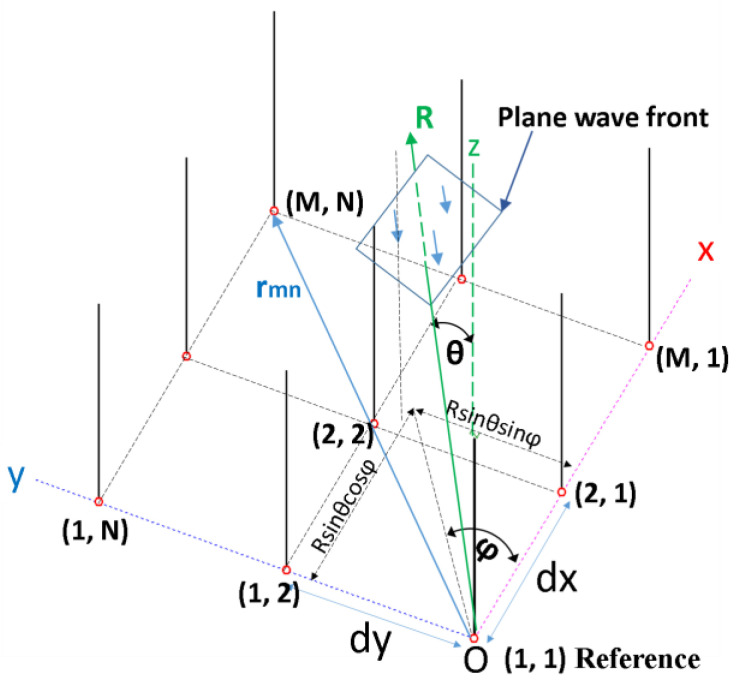
Schematic representation of a rectangular planar antenna array having (M × N) elements.

Generally, the rectangular planar array radiation pattern can be described by the below Equations (1)–(9) [29]. We will consider a rectangular matrix, having M elements on the Ox axis and N elements on the Oy axis (M × N matrix), as represented in Figure 1. The distances between the elements on the Ox axis and those on the Oy were denoted by dx and dy, respectively. Thus, the coordinate of an element of the planar array is (m, n), and its position is indicated by the position vector r_mn_, where 1 ≤ m ≤ M and 1 ≤ n ≤ N. Additionally, since most radiation patterns are represented and studied in the far field (FF), it can be considered that an electromagnetic wave that reaches the array is a plane wave, and its wavefront is perpendicular to its direction of travel, given by R in Figure 1.

The direction of the wavefront was considered to be from the elements far from the origin of the coordinate system, towards the element considered as the reference and having (1, 1) coordinate. Without introducing additional phase shifts besides the natural ones due to the displacement of the electromagnetic wavefront, it can be deduced that between the array elements, the path traveled by the plane wave between an element of coordinates (m, n) and the element of coordinates (1, 1) chosen as reference, is given by (1), and the phase difference between the element situated in (m, n) position, and the element (1, 1) is given by (2), where $k_{0}$ = 2π/λ_0_ represents the free space wave number. Thus, the expression of the radiation pattern of a planar antenna array as a function of the elevation angle *θ* and the azimuth angle *φ* can be written as in (3) [29]. Equation (4) results from substituting the value of $\alpha_{mn}$ from (2) into (3).

We noted with $a_{mn}$ the amplitude of the signal at the supply point of the element (m, n). Since we did not intend to work on the amplitudes and, at the same time, keep this parameter constant for each element and normalized to 1 (in theory, the term used is non-weighted array elements), we assume that $a_{mn}$ = 1 and (4) changes to Equation (5). It was assumed that the elements were identical, and the effects of the mutual coupling between them were neglected. Equation (5) can also be written as a product of two factors, as can be seen in (6), where ${\;F}_{A}\;$ is called the Array Factor and is a function of the geometry of the antenna array and the phase of the excitation signals of the radiating elements, in this case, given the normalization to 1 of the elements’ signals amplitudes.

According to (6), by varying the distances and phase shifts between the array elements, the ${\;F}_{A}$ characteristics and, thereby, the total field of the array, respectively, the radiation pattern, can be controlled. In theory, related to the general case represented in Figure 1, given the sense of the wavefront, which is from the other elements towards the reference element (1, 1), it can be shown that if a phase taper consisting of appropriate values (denoted with $\beta_{mn}$ in Equation (7)) is applied over the elements, the uniform phased planar antenna array should be able to steer its beam to the desired direction $(\theta_{0},\;\varphi_{0}$).

(1) $$\Delta s=(m-1)d_{x}\;\sin(\theta )\cos(\varphi )+(n-1)d_{y}\;\sin(\theta )\sin(\varphi )$$

(2) $$\alpha_{mn}=k_{0}\Delta s=k_{0}(m-1)d_{x}\;\sin(\theta )\cos(\varphi )+k_{0}(n-1)d_{y}\;\sin(\theta )\sin(\varphi )$$

(3) $$E_{tot}\left(\theta ,\varphi \right)=E_{el}\left(\theta ,\;\varphi \right)\sum_{m=1}^{M}\sum_{n=1}^{N}a_{mn}e^{j\alpha_{mn}}$$

(4) $$E_{tot}\left(\theta ,\varphi \right)=E_{el}\left(\theta ,\;\varphi \right)\sum_{m=1}^{M}\sum_{n=1}^{N}a_{mn}e^{j[k_{0}\left(m-1\right)d_{x}sin\left(\theta \right)cos\left(\varphi \right)+k_{0}\left(n-1\right)d_{y}sin\left(\theta \right)sin\left(\varphi \right)]}$$

(5) $$E_{tot}\left(\theta ,\varphi \right)=E_{el}\left(\theta ,\;\varphi \right)\sum_{m=1}^{M}\sum_{n=1}^{N}e^{j{[k}_{0}(m-1)d_{x}sin(\theta )cos(\varphi )+k_{0}(n-1)d_{y}sin(\theta )sin(\varphi )]}$$

(6) $$E_{tot}\left(\theta ,\varphi \right)=E_{el}\left(\theta ,\;\varphi \right){\;F}_{A}\left(\theta ,\;\varphi \right)$$

(7) $$\beta_{mn}=-k_{0}(m-1)d\;\sin(\theta_{0})\cos(\varphi_{0})-k_{0}(n-1)d\;\sin(\theta_{0})\sin(\varphi_{0})$$

(8) $${\;F}_{A}\left(\theta ,\;\phi \right)=\sum_{m=1}^{M}\sum_{n=1}^{N}e^{j{(\alpha }_{mn}+\beta_{mn})}$$

(9) $${\beta \prime }_{mn}=k_{0}(m-1)d\sin(\theta_{0})\cos(\varphi_{0})+k_{0}(n-1)d\sin(\theta_{0})\sin(\varphi_{0})$$

For a four-element square-phased antenna array, the distances *d_x_* and *d_y_* are equal and will be denoted by *d*. Thus, the equation of the phase taper is given by (7) so that the array factor in (3) becomes as in Equation (8). As shown in (2) and (7), $\alpha_{mn}$ parameter depends on the plane wave’s natural orientation, $\left(\theta ,\varphi \right)$ without a phase taper and $\beta_{mn}$ is a function of the desired orientation $\;(\theta_{0},\;\varphi_{0}$) and represents the phase taper to be applied. The maximum for the rectangular planar antenna array radiation pattern will occur for $\left(\theta ,\varphi \right)$ = $(\theta_{0},\;\varphi_{0}$) [29].

In conclusion, from a theoretical point of view, by adjusting the progressive phase shift to each element, the direction of the maximum beam (main lobe) radiated by the phased antenna array system can be controlled. The concept of phase taper refers to gradually changing the phase shift (applying progressive phase shift) between elements of the array to direct the maximum beam in a specific direction.

### 3.2. Phase Shift Signs Effects on the Antenna Array Radiation Pattern Orientation

In our analysis, we considered the displacement of the wavefront from the reference element to the other elements so that the progressive phase shifts that will be introduced, first in simulations and then in the real array, were calculated by inverting the sign for the values in Equation (7).

**Figure 2:**
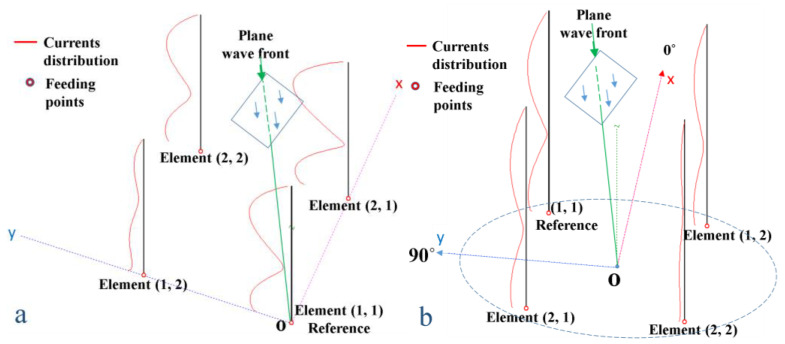
Schematic representation of a square uniform planar antenna array having (2 × 2) elements. Direction of the wavefront was considered to be from elements distant from the origin, towards the element considered as the reference (**a**), respectively from the reference element (1, 1) to the other distant elements (**b**).

This resulted in rotating the radiation pattern by 180° and obtaining the intended results. Numerous simulations were performed using MMANA-GAL, version 3.0.0 [30] antenna design and analyzing software for several beam orientation angles, demonstrating the effect of the sign of the phase taper. Equation (7) was adapted for a square uniform planar antenna array with four elements spaced at half the wavelength corresponding to the frequency of 436.5 MHz (the middle of the 435–438 MHz band) so that M = N = 2, 1 ≤ m ≤ 2 and 1 ≤ n ≤ 2. Figure 2a illustrates the array positioned as in the theoretical aspects shown above. To obtain beam steering considering 0° at the top of the Ox axis and the positive values of the angle as the trigonometric one, the array should need to be physically designed according to the topology in Figure 2b. Thus, in practical experiments, we will make use of the positive phase taper as given in Equation (9), where ${\beta \prime }_{mn}$ is the phase taper used, which has the opposite sign compared to the values of $\beta_{mn}$ in (7).

**Figure 3:**
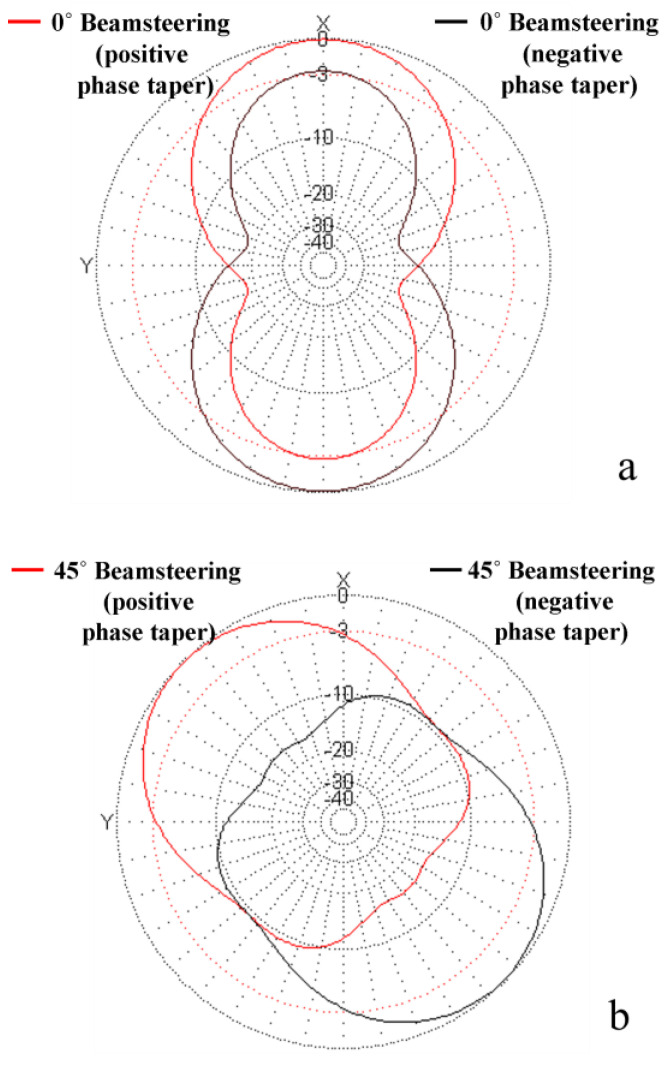
Simulated beam steering for the array topology in Figure 2b, obtained with negative phase taper (plotted in black) according to Equation (7), and positive phase taper (plotted in red) according to Equation (9). The values of $\varphi_{0}$ were 0° in (**a**) and 45° in (**b**), respectively.

Figure 3 shows simulation results corresponding to the topology depicted in Figure 2b, using a negative phase taper (plotted in black in Figure 3) as in (7), positive phase taper (plotted in red in Figure 3) as in (9) and maintaining the same absolute values, corresponding to $\varphi_{0}$ = 0° (a) and $\varphi_{0}$ = 45° (b).

### 3.3. The Phase Shifting Algorithm

After many simulations, it was concluded that for the calculation of the phase shifts that must be introduced to each element of the Phased Antenna Array System to orient the beam in the desired direction in the azimuthal plane, it was considered necessary to establish an optimal constant elevation angle (θ_0_), so that functions which theoretically describe these phase shifts to be dependent only on the azimuth angle, $\varphi_{0}$.

The developed numerical evaluations demonstrated that the most accurate orientation towards the desired angle is achieved for the elevation angle kept at the value of 58.5°, for which the antenna array has a maximum gain of approximative 8 dBi without introducing any phase shifts between its elements. In this case, the directivity characteristic is almost omnidirectional, and as will be shown, the gain is reduced when compared to the scenario where the maximum is oriented according to the angle $\varphi_{0}$. It was found that decreasing the value of $\theta_{0}$ (lower than 58.5°) leads to a decrease in gain and an increase in HPBW (Half Power Beamwidth). If the value is increased, the shape of the radiation pattern is distorted by increasing the magnitude of the secondary radiation lobes.

Adapting Equation (9) for the particular case of a phased square uniform planar antenna array, we arrived at the expressions of the phase shifts that must be applied to each element. If we denote by w1, w2, w3, and w4, the phase shifts introduced to the elements with the coordinates (1, 1), (2, 1), (2, 2), respectively (1, 2), according to Figure 2b, for the orientation of the main lobes on the direction $\varphi_{0}$, the corresponding values are given by:

w1 = 0 (10)

(11) $$w2=\pi \;sin(\theta_{0})cos(\varphi_{0})$$

(12) $$w3=\pi \;sin(\theta_{0})\left[cos\left(\varphi_{0}\right)+sin(\varphi_{0})\right]$$

(13) $$w4=\pi \;sin(\theta_{0})sin(\varphi_{0})$$

Additionally, after calculating the constant $\pi \;sin\left(\theta_{0}\right),$ in which $\theta_{0}$ was 58.5° the resulting value was rounded to 154 and was applied for any beam steering angle $\varphi_{0}$, in the azimuthal plane. The equations shown in (10)–(13) will be used for programming the phased antenna array for different orientation angles of the main lobe. Referring to the element with coordinates (1, 1), the phase shift introduced to its feeding signal was maintained at w1 = 0 regardless of the main lobe steering angle of the phased antenna array because it was set as the reference element. The orientation angle of the main lobe depends on these values (w1, w2, w3, w4), which basically represent the phase shifts that must be introduced to the feeding signals of each element in order to achieve the orientation in the desired direction ($\varphi_{0}$).

This orientation will be determined relative to the position of the LEO satellite at a given time, provided that its azimuth angle from the observer’s ground location is known at that time. Azimuth positions of LEO satellites can be provided by one of the free satellite tracking software (e.g., Orbitron, version 3.71 [31]). The developed numerical algorithm can achieve a high level of performance, needing reduced computational resources.

## 4. Controlled Reception of LEO Satellites Using the Proposed Phased Antenna Array System

**Figure 4:**
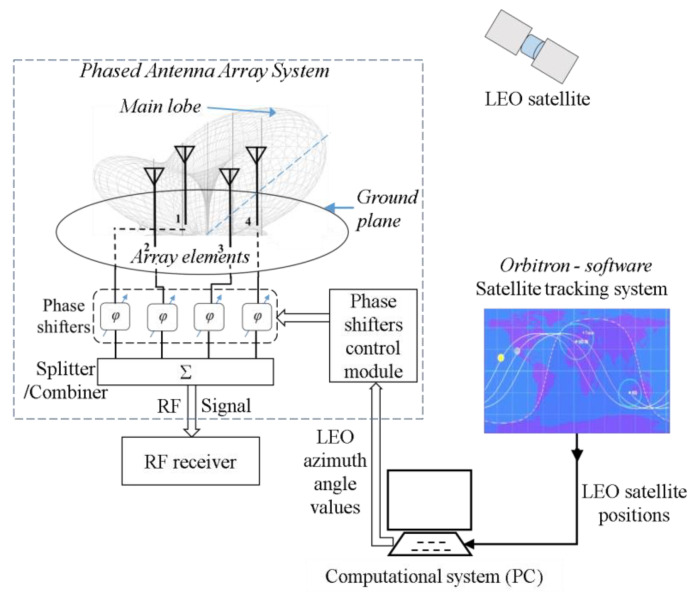
The principle of controlled reception of LEO satellites using the proposed Phased Antenna Array System. The four radiant elements of the phased antenna array system were noted from 1 to 4 (on the left side of the figure).

Figure 4 shows a simplified schematic representation of the principle of controlled reception of a certain LEO satellite, using the potential of the proposed phased antenna array system for this specific application, further demonstrated in this article. In its current state, this system represents an innovative concept whose functionality has been demonstrated in this article and which is ready to be implemented in similar reception systems. To ensure clarity, we have delimited the proposed phased antenna array system with a dotted rectangular outline so as to distinguish it clearly from the other elements in the reception chain. The following segment will succinctly explain the operational mechanism of a reception system based on the simplified depiction in Figure 4.

The signals received by each element of the array are phase-shifted in a controlled manner and then summed. The resulting RF signal is used by the ground station to receive information transmitted using LEO satellites. Certain values of the phase shifts of the elements correspond to a certain angle of main lobe orientation in the azimuthal plane. By changing the phase shifts, the computational system can almost instantly change that orientation angle. In Figure 4, the main lobe provided using the phased antenna array is illustrated. In the vertical plane, the array has the maximum gain in the medium elevation range, which is favorable for the LEO satellite reception, and in the azimuth plane, it is oriented towards one of the satellites. An analysis of the phased antenna array’s radiation pattern in a vertical plane is provided in the next section.

Orbitron [31] is one of the free satellite tracking systems for satellite communication users, weather professionals, astronomers, astrophysicists, and many other users. The azimuth angle values of the satellite provided using the Orbitron software in real-time can be input into the developed control software which further controls the module of the phased antenna array’s main radiation lobe orientation angle. Based on the satellite azimuth ($\varphi_{0}$), which can be imported from the satellite tracking system; the values of the phase shifts (w1, w2, w3, w4) can be derived according to Equations (10)–(13) and then applied to the signals received by the four elements. Thus, the main lobe of the antenna array can be oriented to this azimuth angle.

In the figure shown above, the term Computational system refers to a more general computing system, such as SBC (Single Board Computer) type Raspberry Pi, STM32MP1 (produced by STMicroelectronics, a multinational corporation with headquarters in Plan-les-Ouates, Switzerland ) can also be used. This system runs the satellite tracking software (that provides real-time azimuth angle values of the LEO satellites) and also runs the software for controlling the main lobe orientation of the phased antenna array system and integrates the azimuth angle values to track the LEO satellites. The computed values are provided to the phase shifters control module in binary format. Regarding the phased antenna array system itself, which is the main subject of this article, we will briefly present its component elements in Section 5.

The Phase shifters control module is based on a Teensy 4.0 [32] development board that contains the ARM Cortex-M7 processor, which offers significantly higher operating speed compared to conventional 32-bit processors. Its primary function is to receive binary values provided by the computer via a USB connection and serially transmit them to the phase shifters, through which the phase shifts of the supply signals of the four elements of the phased antenna array are introduced. The signals from the phase shifters were summed using a “Minicircuits” splitter/combiner. The phase shifters utilized were integrated vector modulator-based, and their basic operation mode will be described in Section 5. Finally, the antenna array itself consists of four vertical monopole antenna elements configured at three-quarter wavelength, arranged in a square topology (with each element positioned at the corners of the square). This will also be discussed in the following section.

## 5. Design and Implementation of the Phased Antenna Array System for Leo Satellite Communications

The construction of an antenna array typically begins with selecting the frequency band and configuring the elements to be integrated into it, tailored to meet specific application requirements. In this case, these factors were elaborated upon in Section 2, where the rationale for choosing the (435–438) MHz frequency band and elements with favorable characteristics for medium elevation angles was described, leading to the proposal of utilizing the capabilities offered by elements configured at three-quarter wavelength.

To emphasize this directivity characteristics favorability of the three-quarter wavelength element, we conducted an overlay comparison simulation between the proposed three-quarter wavelength and the commonly used quarter wavelength element configurations.

**Figure 5:**
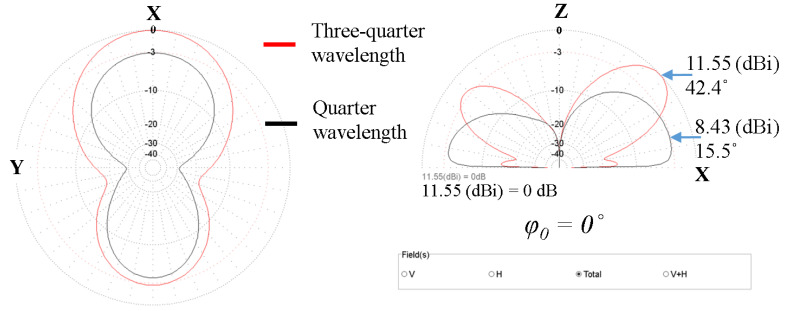
Superimposed simulation results on a four−element quarter wavelength (in black) and three−quarter wavelength (in red) phased uniform planar antenna arrays in horizontal plane xOy (**left**) and in the vertical plane xOz (**right**), corresponding to 0° beam steering angle.

As described in Section 2, during low elevations (below 25°), satellite reception is impacted by atmospheric absorption, interference, and reflections, particularly noticeable at considerable distances from the reception system. Furthermore, at high elevations (above 67°), the Doppler effect assumes significance due to the progressively higher velocities of the satellites. Additionally, it’s noteworthy that satellite passes are rare. So, the medium elevation range offers a balance between good visibility of LEO satellites and the distance above the horizon, so signal reception can be done in more favorable conditions. Figure 5 shows the superimposed simulation results on widely used four-element quarter wavelength and three-quarter wavelength phased antenna arrays. We can observe that between 28° and 61° elevations, the three-quarter wavelength array’s maximum gain is clearly higher (11.55 dBi) than that of the quarter-wavelength array (8.43 dBi). The representations are in the horizontal plane xOy (left) and in the vertical plane xOz (right), corresponding to 0° beam steering angle. Additionally, simulations show that the elevation angle limits corresponding to the maximum value of the main lobes vary between 42.5° (for 0° azimuth angle) and 43° (for 45°, 150°, and 210° azimuth angles). The simulated gain for the other beam steering angles different from 0° was about 11.97 dBi.

**Figure 6:**
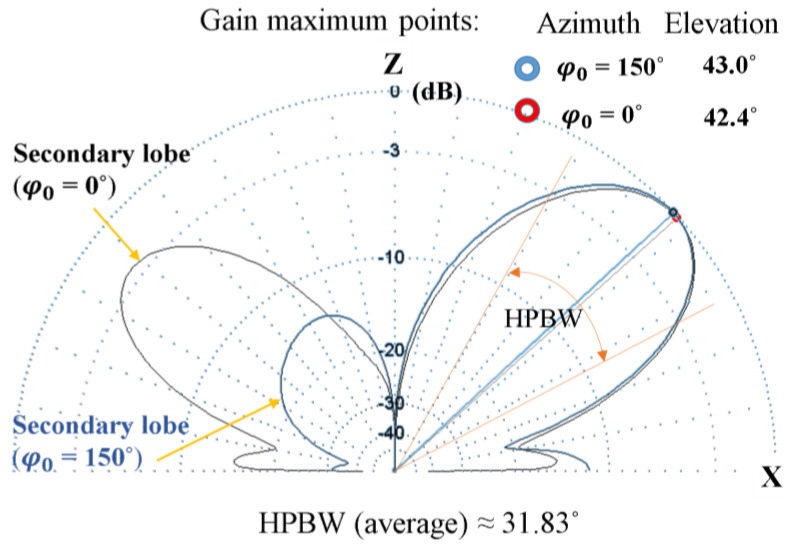
Simulated radiation patterns of three-quarter wavelength phased uniform planar antenna array for 0° and 150° azimuthal beam steering angles.

Figure 6 shows the simulated radiation patterns of the proposed phased antenna array for azimuth beam pointing angles of 0° and 150°. It can be observed the presence of secondary radiation lobes in the vertical plane, which have the greatest prominence for the azimuthal beam steering angle of 0°, with the difference between the maximum gain corresponding to the main lobe and that of the secondary lobes being approximately −2.3 dB. For the other azimuthal orientation angles, the levels of the secondary lobes are insignificant, having an average value of approximately −12.7 dB. This leads us to conclude that the F/B is minimal at the 0° beam steering angle in the azimuth plane and much higher for the other beam steering angles.

After selecting the configuration of the elements for the proposed antenna array, our next focus lies on exploring aspects concerning the definition of these elements. Commencing with preliminary observations derived from simulation results marks the initial step in the following discussion.

Any antenna array is subject to mutual coupling effects between its elements. Taking these into account, it was considered necessary to carry out more simulations on the uniform planar antenna array in order to obtain certain starting points towards its practical realization, including the dimensioning of the antenna elements corresponding to the frequency band of interest, which, in this case, is between 435 and 438 MHz (the band used by many LEO satellites).

As the central frequency introduced in the simulations, the middle of this band was chosen, i.e., 436.5 MHz. The ground plane was made of aluminum sheet, so the values entered in in the simulation model for ground setup were ε = 500 (dielectric constant) and σ = 100,000 mS/m (conductivity). Since the antenna array was intended to be square uniform planar, the elements’ spacing was the same (*d_x_* = *d_y_* in the context of Figure 1). Three-quarters of a wavelength vertical monopole antennas were chosen as antenna array elements, as shown above. We chose a half-wavelength spacing between array elements because the physical phenomena that occurred are easier to manage in the practical case using that distance. Compared to a larger spacing, the number of grating lobes is reduced, the gain is higher, and the HPBW is lower, which offers a much more accurate beam steering. If the distance is smaller than λ/2, the gain decreases, and the unwanted effects of mutual coupling on impedance, resonant frequency, and SWR (Standing Wave Ratio) increase. Additionally, HPBW increases, leading to a decrease in beam steering accuracy [12,29,33].

Due to mutual coupling effects between array elements [12,34], it is expected that the resonant frequency of the system of four antennas will be greater than that of a single element tunned on the same frequency and have the same length. Previous research [26] showed that in practice, for tuning a two-element three-quarter wavelength antenna array to the resonant frequency close to 434 MHz, each individual element needed to be dimensioned for 426 MHz (about 8 MHz lower) resonance. Then, after implementation into the two-element antenna system, fine-tuning of the elements was initiated while keeping the same length corresponding to 426 MHz.

In the case of the four-element array, we initially dimensioned one of the identical elements to be three-quarters of the wavelength, corresponding to the frequency of interest of 436.5 MHz, resulting in a length of approximately 51.52 cm. Afterward, the other three elements were added to form the complete antenna array, and the resonant frequency of the array increased to approximately 460 MHz due to the mutual coupling effects. All elements were considered identical and spaced at λ/2. Subsequently, we utilized the optimization feature available in MMANA-GAL to fine-tune the lengths of the elements incrementally until achieving the desired resonance frequency of the array to approximately 436.5 MHz. As a result, the length of each element increased to approximately 54.31 cm. This procedure was essential to provide us with a starting point in the physical construction of the array of elements and to evaluate the resonant frequency that each element should be tuned to. Following additional simulations, it was ultimately determined that each element should be tuned to approximately 406 MHz, with a length close to 54.31 cm. The simulation results showed that the impedance was close to the desired value of about 50 Ohms, and the SWR was nearly ideal (≈1.0). Additionally, the simulation indicated that the gain of each individual element was approximately 6.5 dBi (decibels relative to isotropic), and the Front-to-Back ratio (F/B) was close to 0.

Thus, considering the definition of the elements based on the simulation results, the next stage involves the practical implementation of the antenna array, which will be further elaborated. In order to minimize signal reflections in the entire array as a system, it was aimed to obtain a SWR as low as possible. The proposed phased antenna array has a ground plane as a mechanical base, which constitutes the border between its two main parts: the radiating part (which includes radiant elements placed above the ground plane) and the hardware part, placed beneath the ground plane and containing passive components and hardware electronics for the beam steering function. For the array radiating elements, four telescopic antennas, on which SMA (Amphenol) connectors were fixed by tinning, resulting in four almost identical elements.

**Figure 7:**
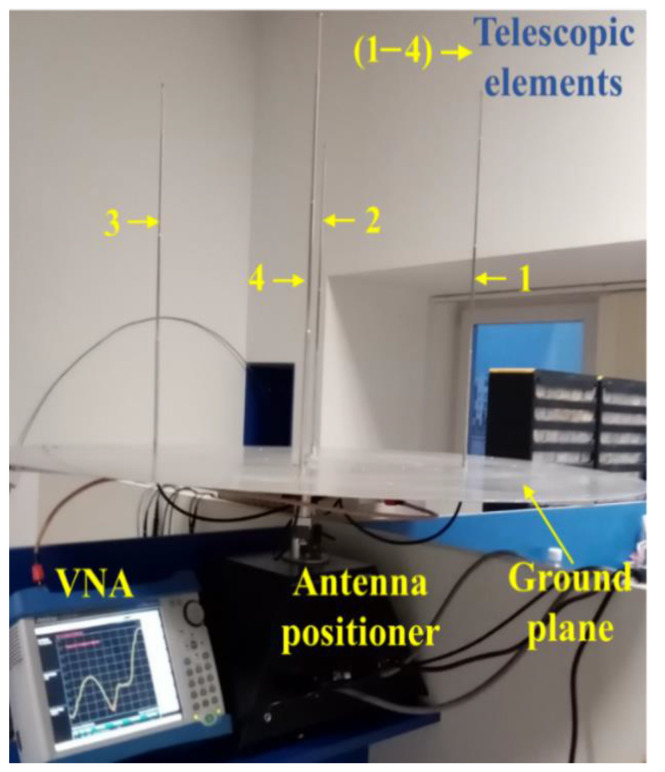
Setup for preliminary measurements on the four-element uniform planar antenna array (radiating part) during the process of fine-tuning the element lengths for the resonant frequency.

The ground plane was made of an aluminum sheet with a thickness of 1.5 mm, processed in a discoidal shape with a diameter of 1 m, large enough for the distance from the elements to the edge of the plane to be greater than or equal to λ/4 (minimal condition for the ground plane). The metallic ground plane serves as a reflecting surface for electromagnetic waves and a reference for the antenna elements. It also plays a crucial role in the antenna array’s overall performance. Before fixing all four elements, each one was fixed on the ground plane and tuned individually to about 406 MHz (as discussed above). The successive measurements on each element were performed with a VNA (Vector Network Analyzer), Anritsu MS8212E series (Atsugi-shi, Japan). The resonant frequency was approx. 406 MHz and the corresponding SWR was close to 1.00 (ideal) for each of the four elements. Additionally, the element impedances were very close to 50 Ohm, a very important fact for impedance matching with the other hardware components, which are designed to have 50 Ohm impedance as well. Once the procedures described above have been completed, for the array resonance fine-tuning, we moved on to fixing the elements on the ground plane to form the planar antenna array, which constitutes the radiant part of the phased antenna array. After the fine-tuning, a resonance frequency close to 436.5 MHz was obtained. A picture of the setup for preliminary measurements can be seen in Figure 7.

To ensure good mechanical stability during the measuring process, the array was placed on the antenna positioner, which is part of the Lab-Volt equipment that will be used to obtain the radiation pattern. A Minicircuits power splitter/combiner [35] was used to combine the signals provided by the antenna elements. The transmission lines (RG058) had the same length of about 35 cm. The VNA was coupled at the combiner input.

**Figure 8:**
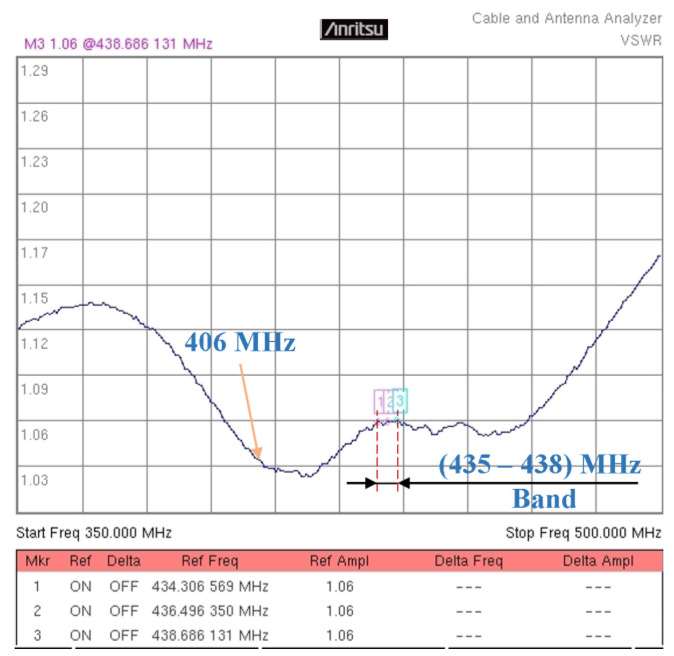
VSWR measurement results were reached after the fine-tuning of the array. The markers 1, 2, and 3 indicate the same VSWR of 1.06 in the frequency band (435–438) MHz.

Figure 8 shows the VSWR measurement results obtained after the fine-tuning of the four-element array. A lower VSWR than that corresponding to the frequency of 436.6 MHz can be observed. This is due to the resonance of each individual element, close to 406 MHz. The effects of mutual coupling between the elements determine a resonance at a higher frequency. In this case, in the band of interest between 435 MHz and 438 MHz, the VSWR has a very good value of 1.06.

The proposed design was thought of as a compact system prepared for LEO satellite reception applications, even in outdoor conditions for IoT application scenarios [36,37]. Thus, the entire hardware was fixed on the ground plane at the opposite part containing the radiating elements. The use of this concept offers the minimization of common mode currents and environmental influence factors.

**Figure 9:**
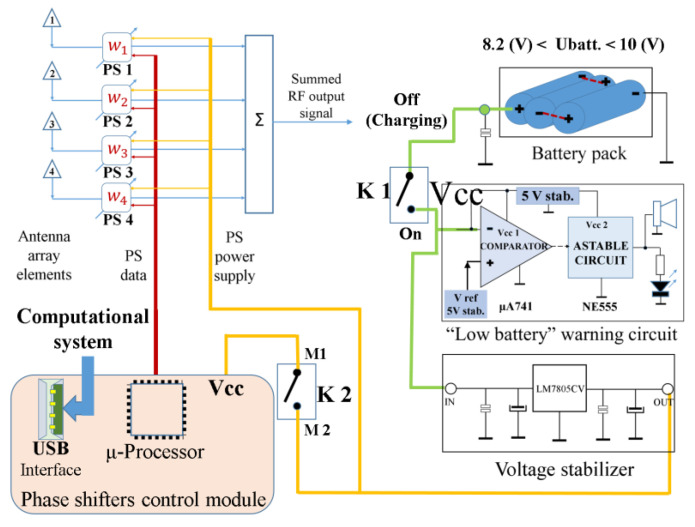
Simplified block diagram of the proposed Phased Antenna Array.

Figure 9 provides a simplified block diagram of the proposed Phased Antenna Array System. The four elements of the antenna array (on the left side of Figure 9, numbered from 1 to 4) constitute the radiating part. The phase shifters (denoted with PS1 to PS4), Phase shifters control module, the splitter/combiner (used as a signal adder and marked with “Σ”), Battery pack, “Low battery” warning circuit, and Voltage stabilizer constitute the hardware part, these components being placed below the radiating part of the antenna array (below the ground plane).

The phase shifters are denoted with PS1–PS4, and the corresponding phase shifts are introduced with *W*_1_*–W*_4_. All components were electromagnetically shielded and electrically connected to the ground plane.

**Figure 10:**
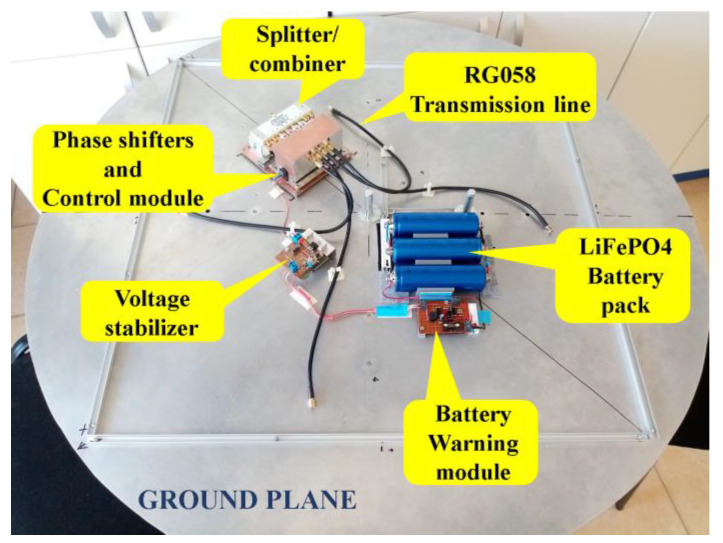
The hardware of the Phased Antenna Array System (passive components and hardware electronics which were fixed on the ground plane, at the opposite part with that containing the radiating elements).

An image from beneath the ground plane, opposite to the location where the vertical antenna elements of the array have been fixed, is depicted in Figure 10. This image illustrates the positions of each hardware component within the proposed phased antenna array. Each component will be presented below.

A module for controlling the shape and direction of the antenna array radiation pattern (also generally known as a beamforming module [12,26,33]) was designed and implemented and consists of a splitter/combiner, phase shifters, basic PCB and microprocessor-based control module. In the phased antenna array presented in this article, we have successfully integrated the original concept together with previously developed phase shifters that have already been tested for linear antenna arrays.

The phase shifters utilized were equipped with AD8340 [27] integrated circuits produced using Analog Devices. This circuit, in order to achieve the desired phase shifts, requires two differential voltages to control the phase imposed on the RF input signal. Their values are normally included in the range of −500 mV to 500 mV in common mode. To obtain those values with good accuracy, each of the phase shifters was equipped with a DAC8554 [28] that contains four digital-to-analog converters (DAC), each with a 16-bit resolution (65,536 stages of voltage variation for each DAC). The operation mode of the control module and phase shifters command software is presented below.

Each of the four-phase shifters available and tested in previous research is equipped with the following essential components: a DAC8554 circuit, an AD8340 vector modulator circuit, and an LM4140 integrated circuit developed by National Instruments, with the role of voltage source used as a reference for DACs. To achieve a certain phase shift, the AD8340 circuit requires two differential voltages, which we denoted by *V_x_* and *V_y_*. These voltages are functions of the phase shift that a phase shifter will achieve. For the orientation of the main lobe of the phased antenna array, we started from the results obtained in (10)–(13), which led to obtaining four values (w1, w2, w3, w4) for each chosen angle, denoted by $\varphi_{0}$. Each of the “wi” values (“i” having integer values between 1 and 4) represents the phase shift that needs to be achieved using one of the four-phase shifters. Then, analog quantities *V_x_* and *V_y_* were obtained, and based on them, the software calculated the pairs of values that form the differential voltages. These were denoted by *V_x_+*, *V_x_*−, *V_y_*+, and *V_y_*−. Thus, the difference between *V_x_*+ and *V_x_*− is equal to the differential voltage *V_x_*, and the difference between *V_y_*+ and *V_y_*− is equal to *V_y_*. After converting those pairs of values to binary numbers, they were transmitted serially (via USB) from the computational system to the control hardware module, which in turn transmitted them to each phase shifter in order to be written on the four DACs. The same procedure was applied for each of the four-phase shifters that will feed each element of the antenna array. After the conversion from digital to analog, the differential voltages were obtained to command the AD8340 circuit to achieve the phase shift of the RF signal. The high-performance Teensy 4.0 development module included an ARM Cortex-M7 at 600 MHz microcontroller with the possibility of overclocking up to 1 GHz, float point math unit—64 and 32 bits, 1984K Flash, 1024K RAM, USB device 480 Mbit/sec and USB host 480 Mbit/sec was used for controlling the phased antenna array’s main radiation lobe orientation in the azimuth plane. As shown in Section 4, the data regarding the satellite azimuth angle can be imported from Orbitron software, the phase shift values (w1, w2, w3, w4) given by (10)–(13) and applied to the signals received by the four antenna array elements being dependent on that azimuth angle, $\varphi_{0}$.

**Figure 11:**
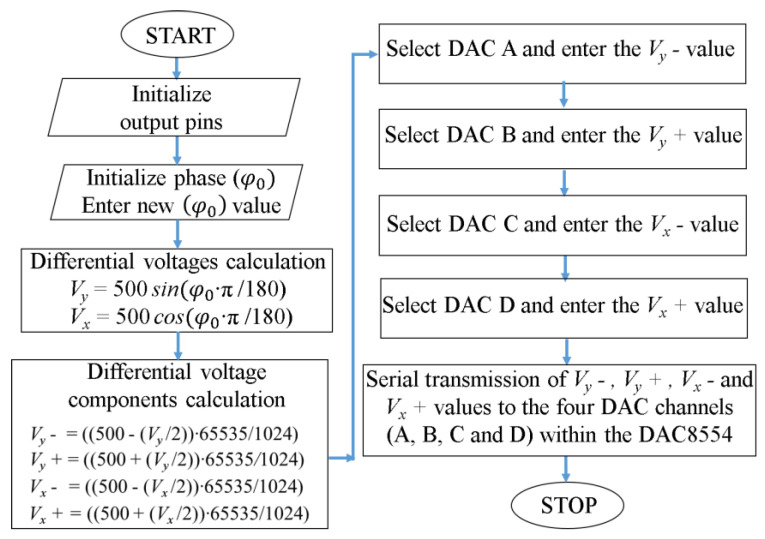
The logical diagram of phase shifters control software.

The phase shifters control software was written using the C++ programming language, extended with the Teensy 4.0 specific add-on. Although the AD8340 circuit has the ability to adjust the signal amplitude as well, this facility has not been used, keeping the attenuation to a minimum to give the antenna array maximum sensitivity. Figure 11 can be seen as a simplified diagram of how the software works for programming a phase shifter. The programming of each of the four-phase shifters was based on the same software diagram.

**Figure 12:**
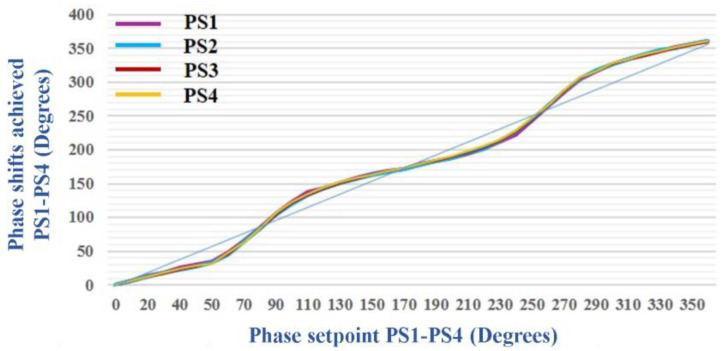
The phase shifts achieved by the four-phase shifters (PS1–PS4) are to be implemented in the phased antenna array. The measurements were performed at a frequency of 435 MHz.

The phase shifters were previously tested within linear antenna arrays in the 868 MHz and 915 MHz frequency bands [38,39]. The frequency range for the AD8340 vector modulator circuit is 700 MHz–1 GHz. Given the availability of these phase shifters, in the idea of using them for a lower frequency range assigned to the reception of LEO satellites, many preliminary operation tests of those were carried out in the 435 MHz band. Figure 12 shows the results obtained from these laboratory tests.

**Figure 13:**
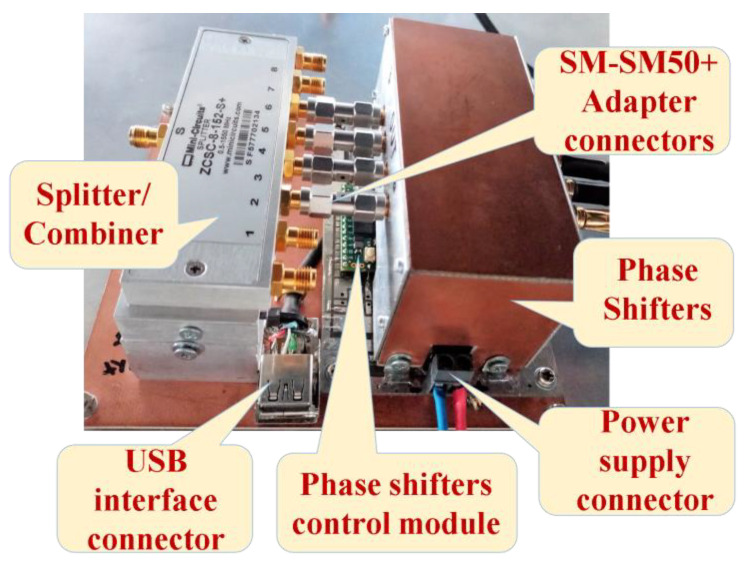
Close-up view of the phase shifters control module, splitter/combiner, phase shifters and USB programming interface, integrated on the same PCB.

The phase shifts introduced by the four-phase shifters (PS1–PS4) implemented in the phased antenna array had very close values for the same phase setpoints. The graph of Phase shifts vs. Phase setpoints indicates an acceptable correspondence between the phases achieved by the PSs and the phase setpoints, being close to the graphs for (700 MHz–1 GHz) in the AD8340 datasheet. The module can be seen in Figure 13. The “Minicircuits” splitter/combiner shows linear characteristics in a very wide frequency band, including the one of interest to us, located between 435 MHz and 438 MHz [35].

In order to make the signal processing part more compact, a module containing the phase shifters, the phase shifters control module, the splitter/combiner, and the USB programming interface were developed. In designing and making the PCB needed for this module, the distance between the component elements and the need to make an electromagnetic shield for the phase shifters were taken into account. To minimize signal attenuation and introduce additional phase shifts, SM-SM50+ adapter connectors were used to connect the four outputs of the phase shifter to the inputs of the combiner.

The power supply system was initially thought to provide autonomy in the process of measuring the radiation pattern of the antenna array so that there was no need for extra power wires that could disturb the rotation movement of the Lab-Volt antenna positioner during the radiation pattern measurements. The battery-based power supply uses LiFePO4 technology, which requires no maintenance, allows for quick charging, and is environmentally friendly.

## 6. Performance Evaluation

The measuring radiation pattern setup of the phased antenna array contained the emission part and the reception part. The phased antenna array represented the AUT (Array Under Test) and was installed in reception mode. A Yagi-Uda antenna was used in transmit mode as a test antenna for the AUT.

**Figure 14:**
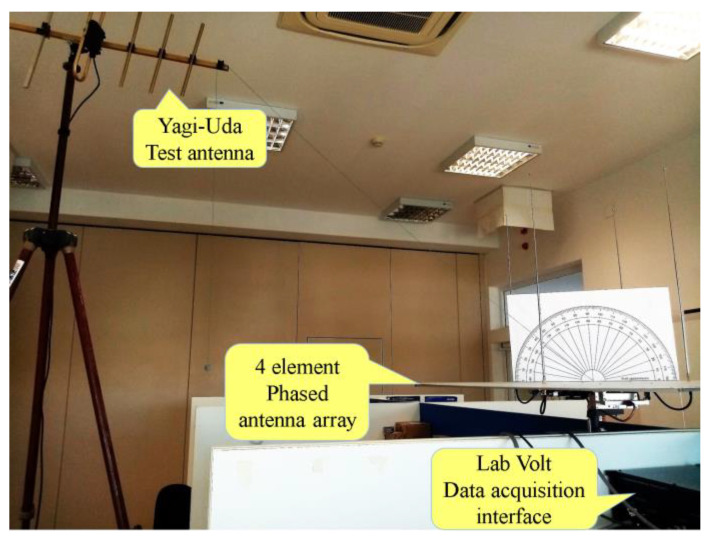
The measuring radiation pattern setup of the four-element Phased Antenna Array System. Before starting the measurements, the protractor was used to adjust the elevation to 42°.

As can be seen in Figure 14, for this antenna, a fixing system was adapted on a tripod with various mechanical adjustment facilities so that the test antenna could be fixed in various positions relative to the phased antenna array concerning height, axis tilt, and rotation around the tripod axis. An Agilent N5182A (MXG Vector Signal Generator) RF generator was configured at a frequency of 436.5 MHz and used as a sinusoidal signal generator, amplitude modulated with a 1 KHz sinusoidal signal. A low-loss RG058 transmission line with 50 Ohms impedance was used between the RF generator and the Yagi-Uda antenna.

During the experimental measurements, the generator was adjusted to the output power of −2.3 dBm, and the RG058 transmission line was approximately 15 m long. Consequently, the power of the Yagi-Uda antenna feed signal was approximately −7.3 dBm. The ground station receivers can usually detect signals with an MDS level (Minimum Discernible Signal) between approximately −95 dBm and −138 dBm [40]. According to the simulation results, the proposed phased antenna array has a high gain, approximately between 11.55 dBi and 11.97 dBi, for different main radiation lobe orientation angles. These results demonstrate that the developed array system is suitable for implementation in LEO satellite reception systems.

**Figure 15:**
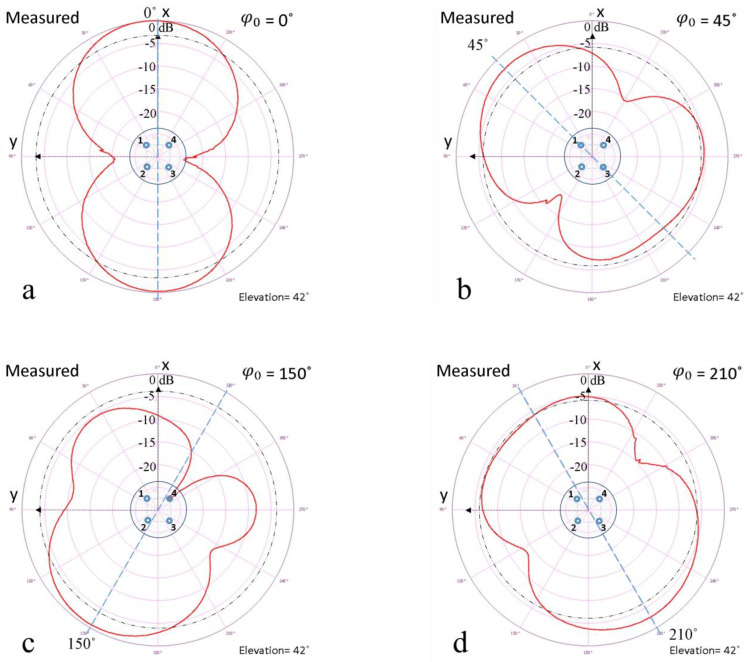
Experimental results depicting the radiation patterns of the proposed phased antenna array system for azimuthal beam steering angles of 0° (**a**), 45° (**b**), 150° (**c**) and 210° (**d**). The circle within each diagram represents the antenna array with its four elements, numbered from 1 to 4.

In Figure 14, it can be seen that the Yagi-Uda test antenna was positioned for radiation pattern measurements corresponding to approximately 42° elevation, so its axis was along the Ox axis of the phased antenna array under test. This led to a separation of about 1.5 m between the center of the array’s ground plane and the Yagi-Uda antenna tip. That was considered as the axis that passes through the center of the ground plane of the array and parallel to the imaginary lines that join elements 1 and 2, respectively, 3 and 4. The positions of the array elements, thus numbered, are shown in the images of experimental results on the radiation patterns revealed in Figure 15, where the phased antenna array system was symbolized by a circle, and its elements were numbered from 1 to 4. To obtain the phased antenna array radiation patterns, the Lab-Volt laboratory equipment was used, which included the antenna positioner and the data acquisition interface. The signal received by the antenna array was amplified by the input amplifier included in the antenna positioner and then sent to the data acquisition interface.

The ground plane of the antenna array was provided with a fixing system perfectly adapted to that of the antenna positioner for the safe fixing of the entire system that formed the array. At each measurement of the radiation pattern, the positioner, together with the antenna array fixed on it, performed a full rotation movement of 360° around the center of the discoidal ground plane of the array. The signals received by the antenna array were then amplified and processed by the data acquisition interface. Via the specialized software of the Lab-Volt equipment, the signals were processed and recorded to display the radiation patterns with a 1° resolution in the horizontal (azimuth) plane.

Measurement results of the phased antenna array radiation patterns are shown in Figure 15. For clarity, in each of Figure 15a–d, the main lobe orientation angle, $\varphi_{0}$ is specified.

Generally, in the analysis of simulation results, the HPBW of a main lobe can be determined by estimating the value of the central angle, whose segments are on either side of the maximum point of the lobe and intersect the auxiliary circle corresponding to the gain of −3 dB which has a constant diameter for any orientation angle of the lobe, because the software automatically normalizes the radiation patterns to 0 dB, regardless of the beam steering angle. In real measurements, the auxiliary circles for HPBW determination were depicted with dotted black lines and corresponded to 3 dB lower gain values than those corresponding to a maximum value of the main lobes for each beam steering angle.

After estimating the HPBW in simulations, a constant value of 80° was observed. Measurement results presented in Figure 15 indicated a minimum HPBW of 76° corresponding to $\varphi_{0}$ = 0°, a maximum of 87° for $\varphi_{0}$ = 210°, and for the other orientation angles, it was close to 78°. Following these, it can be observed that there is a good agreement between the simulation and measurement results of HPBW. However, regarding measurement results, some differences can be observed in the HPBW for various orientation angles, primarily due to technical imperfections and the indoor measurement environment. It is expected that in LEO satellite reception in the outdoor environment, the differences between the HPBW values for different beam steering angles will be less than or equal to 80°. With all these differences, for an antenna array designed to receive LEO satellites in the 435–438 MHz frequency band, even an HPBW between 62° and 87° may be adequate because it can provide relatively wide coverage of the passage area of satellites that move rapidly in their orbits.

At the same time, the possibility of electronically controlled beam steering of the antenna array can be useful in tracking the satellites we want to receive signals from while providing a better SNR (Signal-to-Noise Ratio) than in the usual case of omnidirectional antennas for reception of LEO satellites and also a more stable and robust connection (good connection in the presence of various obstacles or under the interference effects) with satellites during their passes.

### 6.1. Comparative Examples

**Figure 16:**
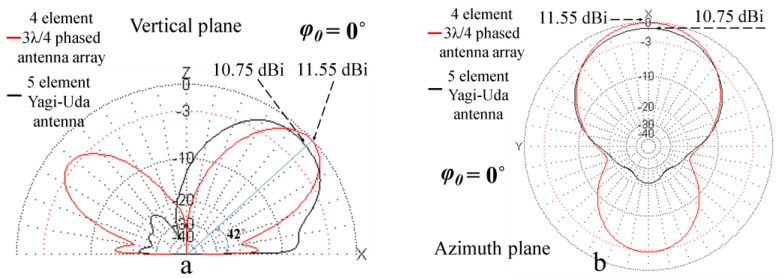
Comparison of simulated radiation patterns for a 5-element Yagi-Uda antenna (plotted in black) and the proposed phased antenna array (plotted in red) in the vertical plane (**a**) and the azimuth plane (**b**), corresponding to 0° azimuthal beam steering angle.

The proposed four-element, three-quarter wavelength antenna array is equivalent in terms of polarization, main lobe gain, radiation pattern, and beam steering to a five-element vertical polarized Yagi-Uda antenna, tilted at an angle of 42°, mounted on an azimuthal antenna rotator. The compared far-field radiation patterns of the two antennas in the vertical plane (a) and in the azimuth plane (b) are shown in Figure 16.

As can be seen, the 5-element Yagi-Uda antenna has a maximum gain of approximately 10.75 dBi. The proposed phased antenna array has a maximum gain close to 12 dBi (the range was approximately from 11.55 dBi at 0° beam steering angle to 11.97 dBi at other angles), a wider HPBW (62–87°), depending on beam steering angle, and the main lobe orientation can be controlled electronically, without the need for rotators.

Table 1 highlights the comparison between the qualitative aspects of the systems widely used in LEO communications in terms of the phase-shifting techniques used, the complexity of the associated systems, and the types of polarization considered. In light of the analysis presented in Table 1, it is distinguished that the proposed phased antenna array stands out for its high beam direction resolution and fast signal processing capabilities. These characteristics are favored by the use of the phase shift technique based on integrated vector modulators. It is also found that the complexity and cost of the system are reduced compared to other similar solutions, which do not offer significant advantages in terms of azimuth scanning capability and satellite tracking speed for low-orbit (LEO) satellite communications reception. These aspects are relevant in the context where the proposed system achieves a balanced compromise between complexity and performance. The analysis of the table reveals that the proposed system offers several advantages, including low processing power requirements, system simplicity, the capability to orient the main lobe to any angle within the range of 0° to 360° with high angular resolution (less than 1 degree), and very low delays in signal processing. Regarding the type of polarization, circularly polarized elements are used more often in satellite communications for certain reasons. First of all, the transmitted signal is less affected by changes in the orientation of the satellite. Additionally, it provides satisfactory robustness against fading and interference, which can improve performance in noisy or heavily spatially multiplexed environments. This polarization also allows better isolation between transmitted and received signals, thus reducing cross-channel interference. However, the disadvantages of using circularly polarized elements in phased antenna arrays for LEO satellite reception can involve increased complexity, additional signal loss, and higher costs. On the other hand, the three-quarter wavelength, vertically polarized elements selected for the proposed phased antenna array offer a significantly higher gain, coupled with improved directivity and increased resistance to interference and fading. In Table 1, we note that delay line-based phase shifting has limited angular beam steering resolution due to its predefined structure for phase shifting. This also entails relatively complex design requirements. Additionally, turnstile-type elements chosen by Ibelings [19] are complex, necessitating meticulous management of numerous variables for precise main lobe orientation within the array. The coherent SDR technique for phase shifting, although it provides a high angular beam-steering resolution and simultaneous multi-satellite tracking, these benefits can be achieved at high costs and by using very high processing power, but a significant disadvantage is represented by substantial delays in processed signals, caused by factors such as system complexity.

### 6.2. Limitations of the Proposed System

To evaluate the performance of the proposed antenna array in terms of Operating Frequency, Bandwidth, VSWR, and Return Loss, we can compare the data from Table 2, which was compiled to present these parameters for antennas developed in 2007 [41], 2009 [42] and 2020 [43], suitable for satellite communications at frequencies of 10 GHz, 5.6 GHz, and 1.8 GHz, respectively. Thus, one of the identified limitations of our antenna system results in the smallest measured value of bandwidth (BW), recorded at 87 MHz (24%), indicating a reduced capacity of our antenna system to cover a wider range of frequencies or to support multiple channels simultaneously compared to previously studied antennas.

Several other factors could be acknowledged as potential constraints for our proposed system. Among them, antennas operating at 436.5 MHz may exhibit restricted performance in signal propagation over extended distances due to inherent wave propagation characteristics at this frequency. Moreover, they may demonstrate heightened vulnerability to interference and spectral congestion attributed to the pervasive utilization of the radio spectrum and nearby electronic devices. Furthermore, compatibility challenges might arise with existing equipment or infrastructure, typically optimized for higher frequencies prevalent in 5G networks.

Nevertheless, the VSWR value of the proposed system is the lowest among those obtained for any of the other antenna systems presented in Table 2, indicating a high efficiency of the antenna array across a wide frequency range. This capability can be considered a significant advantage of the system. Maintaining a low level of VSWR over a broad frequency spectrum is crucial for the optimal performance of the four-element, three-quarter wavelength antenna array in various usage scenarios.

## 7. Conclusions

LEO satellite constellations offer a promising solution for enhancing connectivity in remote areas and supporting existing IoT terrestrial communication networks. Traditional antennas used in satellite communications often rely on mechanical systems like rotators to achieve wide coverage in the azimuth plane, leading to maintenance issues and reduced performance in adverse weather. Phased antenna arrays, however, provide electronically controlled directivity without the need for mechanical adjustments, making them suitable candidates for future LEO satellite ground stations.

Our research focused on the definition and implementation of a phased antenna system based on an original concept in order to explore its potential to complement LEO satellite reception systems. Our aim was to provide a cost-effective alternative, born from academic research, without competing directly with expensive commercial solutions. The construction details reflect a conceptual approach at the prototype level. The functionality of the concept has been demonstrated and is ready for integration into LEO satellite reception systems. As far as we know, it is the first time that a four-element, three-quarter wavelength phased antenna array system has been successfully realized with the intention of being optimized for this specific field of communications. Using laboratory measurements, we demonstrated precise control of the array’s main radiation lobe orientation in the azimuth plane. These results confirmed the presence of a single consistent main lobe and highlighted the system’s reliability in covering the directions in the azimuth plane. Previous research on individual antenna elements showed favorable radiation patterns for medium elevation, supporting the suitability of antenna array systems for LEO IoT satellite communications. The proposed phased antenna array operates in the 435–438 MHz frequency band, utilizing a metallic ground plane to enhance overall performance, including the minimization of common mode currents and environmental influence factors. Simulation results indicated a high gain close to 12 dBi, an azimuthal HPBW of 80°, and a vertical HPBW of about 32° in the medium elevation range, making the array suitable for LEO satellite reception also, including IoT applications. Measurements in an indoor environment confirmed a consistent single main lobe, highlighting the system’s reliable coverage across orientations in the azimuth plane. These results show that the developed array system is suitable for implementation in LEO satellite reception for IoT systems. In the azimuth plane, the F/B values close to 6 dB for 45°, 14.5 dB for 150°, and 3.5 dB for 210° confirmed the directionality of the array. The measured azimuthal HPBW between 76° and 87° at an elevation of 42° (approximately the midpoint of medium elevation angles) is adequate for LEO satellite reception.

The proposed phased antenna array system can be used to receive LoRa satellites, many of which use the frequency band of 435–438MHz. Due to the ability to control the main radiation lobe orientation, the proposed system, together with specialized LoRa modules used to decode radio signals received from LoRa LEO satellites, can enhance signal-receiving reliability and expand the coverage capacity of IoT networks. Using the proposed concept, the array can be easily reconfigured for use in the ISM frequency band of 868 MHz. The limits of the signal strength received from the LEO satellites can vary from less than −100 dBm to −80 dBm or even more. The simulated gain of the array was high, ranging between 11.55 dBi and 11.97 dBi. Additionally, the power feeding the Yagi-Uda antenna for radiation pattern measurements was approximately −7 dBm, while the ground station receivers generally demonstrated the capability to detect signals with an MDS level ranging from approximately −95 dBm to −138 dBm. These factors collectively led us to conclude that the proposed phased antenna array is well-suited for LEO satellite communications.

## Figures and Tables

**Table 1:** Comparison of phase shift techniques, advantages, and disadvantages of different phased antenna array systems concepts.

	Antenna System	Phase Shift Technique Basis	Advantages	Disadvantages
**Proposed system**	Three-quarter wavelength antenna array	Vector modulator	▪ Requires low processing power ▪ System simplicity ▪ High angular beam steering resolution ▪ Very low delays in processed signals	▪ Linear polarization ▪ Sequential satellite tracking
**Ibelings** **[19]**	Turnstile antenna array	Delay lines	▪ Requires low processing power ▪ Circular polarization	▪ Low angular beam steering resolution ▪ High complexity of the delay line network and antenna elements ▪ Sequential satellite tracking
**Kraken** **SDR** **[21,22]**	Whip antenna array	Coherent SDR’s	▪ High angular beam steering resolution ▪ Simultaneous multi-satellite tracking	▪ Requires very high processing power ▪ Substantial delays in processed signals

**Table 2:** Operating Frequency, Bandwidth, VSWR, and Return Loss for various antenna types used for satellite communications.

	Antenna System	Operating Frequency	−10 dB Bandwidth (MHz)	VSWR/ Return Loss (dB)
**Proposed system**	Three-quarter wavelength antenna array	≈436.5 MHz	≈87 (24%)	1.06/ −30.68
**[41]**	Coplanar Waveguide (CPW) fed quasi-Yagi	≈10 GHz	≈4400 (44%)	1.13/ −24.24
**[42]**	Printed quasi-Yagi with Compact Transition	≈5.6 GHz	≈1848 (33%)	1.49/ −14.12
**[43]**	Printed quasi-Yagi with monopole elements	≈1.8 GHz	≈856.8 (47.6%)	1.29/ −17.94

## Data Availability

Data are contained within the article.
